# Association between glycemic traits and melanoma: a mendelian randomization analysis

**DOI:** 10.3389/fgene.2023.1260367

**Published:** 2023-12-20

**Authors:** Yun-Chao Zhang, Cen-Di Lu, Quan-Yao Li, Jin-Na Shi, Jun Shi, Min Yang

**Affiliations:** ^1^ Department of Oncology, Yueyang Hospital of Integrated Traditional Chinese and Western Medicine, Shanghai University of Traditional Chinese Medicine, Shanghai, China; ^2^ Department of Neurosurgery, The Second Affiliated Hospital of Zhejiang Chinese Medical University, Xinhua Hospital of Zhejiang Province, Hangzhou, Zhejiang, China; ^3^ Department of General Practice, KangQiao Campus of the Second Affiliated Hospital of Zhejiang Chinese Medical University, Xinhua Hospital of Zhejiang Province, Hangzhou, Zhejiang, China; ^4^ Department of Traditional Chinese Medicine, Shanghai Fourth People’s Hospital Affiliated to Tongji University School of Medicine, Shanghai, China; ^5^ Department of Oncology, The Second Affiliated Hospital of Zhejiang Chinese Medical University, Xinhua Hospital of Zhejiang Province, Hangzhou, Zhejiang, China

**Keywords:** two-sample mendelian randomization, glycemic traits, melanoma, genome-wide association study, causality

## Abstract

**Background:** The causation of Glycemic Traits and risks of Melanoma remains unknown. We used Mendelian Randomization (MR) to assess the links between Glycemic Traits and Melanoma.

**Method:** Pooled data from Genome-Wide Association Studies (GWAS) were utilized to examine the relationships that exist between Fasting Insulin (n = 26), 2-h Glucose (n = 10), Fasting Glucose (n = 47), HbA1c (n = 68), and Type-2 Diabetes (n = 105) and Melanoma. We evaluated the correlation of these variations with melanoma risk using Two-Samples MR.

**Result:** In the IVW model, Fasting Glucose (OR = 0.99, 95%CI = 0.993–0.998, *p* < 0.05, IVW), Type-2 Diabetes (OR = 0.998, 95%CI = 0.998–0.999, *p* < 0.01, IVW) and HbA1c (OR = 0.19, 95%CI = 0.0415–0.8788, *p* < 0.05, IVW) was causally associated with a lower risk of Melanoma. In all models analyzed, there was no apparent causal relationship between Fasting Insulin and Melanoma risk. There was no obvious causal difference in the IVW analysis of 2-h Glucose and Melanoma, but its *p* < 0.05 in MR Egger (OR = 0.99, 95%CI = 0.9883–0.9984, *p* < 0.05, MR Egger), and the direction was consistent in other MR analyses, suggesting that there may be a causal relationship.

**Conclusion:** The results of this study suggest that a higher risk of Fasting Glucose, Type-2 Diabetes, 2-h Glucose, and HbA1c may be associated with a lower risk of Melanoma. However, no causal relationship between fasting insulin and melanoma was found. These results suggest that pharmacological or lifestyle interventions that regulate plasma glucose levels in the body may be beneficial in the prevention of melanoma.

## 1 Introduction

Melanoma is a malignant tumor produced by the malignant transformation of melanocytes, which has a high probability of local spread and metastatic spread. Studies have shown that its incidence increases linearly in young and middle-aged people aged 25 to 50, and is high in people aged 57 (Carr et al., 2020). It is less common than other types of skin cancer but accounts for 73 percent of skin cancer-related deaths (Gershenwald and Guy, 2016). Studies have shown that in the next 10 years, the incidence and mortality of melanoma will continue to rise (Whiteman et al., 2016). The intervention effect of early surgical treatment and late radiotherapy and chemotherapy on the prognosis of patients is not satisfactory, and the results of several clinical trials have shown that the objective remission rate of patient’s symptoms after treatment is less than 1% (O'Neill et al., 2006; Atzpodien et al., 2008; Bhatia et al., 2012). It is particularly important to look for risk factors to prevent the occurrence of Melanoma.

Several recent studies have shown that obesity is positively associated with the risk of melanoma (Dusingize et al., 2020; Larsson and Burgess, 2021). Obese adults have a higher prevalence of metabolic problems, such as insulin irregularities, hyperglycemia, and Type-2 Diabetes. Some researches has found that Type-2 Diabetes may be associated with an increased risk of Melanoma (Harding et al., 2015; Yuan et al., 2020). However, other cohort studies and case-control studies have found the opposite results (Qi et al., 2014; Malavolti et al., 2017). Previous prospective studies have shown that higher Fasting Glucose is closely related to the occurrence and development of Melanoma (Stattin et al., 2007). Recent studies have demonstrated a positive association between Glycemic Traits and the risk of colorectal cancer (Murphy et al., 2022a). But the MR studies of the associations between various Glycemic Traits and melanoma have not yet been reported.

This study used MR to explore the causal relationship between Glycemic Traits and Melanoma risk. MR is a comparable method to randomization in randomized controlled trials. When parents with two or more pairs of qualities cross when alleles are separated, genes on non-homologous chromosomes operate as free combinations, according to the law of independent assortment. Since germline genetic variation, and the random nature of allelic segregation is fixed at conception, MR analysis is less susceptible to traditional confounding and reverse causation. In this study, we used GWAS related to Fasting Glucose, Fasting Insulin, 2-h Glucose, HbA1c, and Type-2 Diabetes, GWAS data on Melanoma from risk on United Kingdom Biobank cohort study and FinnGen cohort study (Mahajan et al., 2018; Chen et al., 2021). Two-sample MR was used to explore the potential causal influence of the Glycemic Traits on the risk of Melanoma.

## 2 Methods

### 2.1 Study design

MR investigates the link between exposure and illness by employing genetic variation Single Nucleotide Polymorphisms (SNPs) as Instrumental Variables IV). IV was extracted from a disease-specific Genome-Wide Association Studies (GWAS) dataset for this investigation. The IV in this work should fulfill three criteria: there should be a high connection between IV and exposure, IV should only affect the outcomes through exposure, and IV should not have horizontal pleiotropy. Appropriate SNPs for usage as IVs must be strongly linked to malignancy (*p* < 5 × 10^−8^). To ensure independence, SNPs were restricted by low linkage disequilibrium (LD, *r* ^2^ < 0.001, window size = 10,000 kb) using clumping. By MR GWAS data. from different sources were analyzed to assess the causal relationship between glycemic signature and melanoma risk. Assess the strength of IV using the F statistic (F = beta^2^/se^2^), where *β* is the effect size of the allele and SE is the standard error (Feng et al., 2022). If F > 10, the correlation between IV and exposure was considered strong enough to protect the results of MR analysis from weak instrument bias. Meanwhile, it will ensure no confounders like UV radiation, light skin type, the presence of multiple atypical nevi, and a positive family history using the Phenoscanner (http://www.phenoscanner.medschl.cam.ac.uk/phenoscanner) website to have a search over each SNP (Rastrelli et al., 2014; Ugurel and Gutzmer, 2023).

### 2.2 Data source

Glycemic Traits data come from the largest GWAS to date (Glucose And Insulin-related Traits Consortium). A GWAS study of 2 h Glucose, Fasting Glucose, and Fasting Insulin included 63,396 (SNPs= 27,330,879), 200,622 (SNPs= 31,008,728), and 151,013 (SNPs= 29,664,438) participants of European ancestry, respectively (Chen et al., 2021). The GWAS for Type-2 Diabetes included 74,124 individuals with Type-2 Diabetes and 824,006 controls of European ancestry (SNPs= 21,000,000) (Mahajan et al., 2018). The HbA1c GWAS from the United Kingdom biobank (http://www.nealelab.is/uk-biobank) included 361,194 participants of European ancestry (SNPs= 1,048,575). The GWAS of Melanoma were obtained from United Kingdom biobank (https://www.ukbiobank.ac.uk/) and FinnGen (https://www.finngen.fi/en/access_results), the GWAS of United Kingdom biobank included 3,598 patients and 459,335 for controls (SNPs= 9,851,867) of European ancestry, the GWAS of FinnGen included 393 patients and 180,622 controls (SNPs= 16,380,337) of European ancestry. All study participants gave written informed consent, and the ethics committee approved all studies. SNPs data can be found in Supplementary Table S1.

### 2.3 Method selection

We estimated the relationship between Glycemic Traits and Melanoma risk using MR Egger, Inverse Variance Weighting (IVW), weighted Median, Simple Mode, and Weighted Mode MR methods. The IVW method assumes that all SNPs do not have horizontal pleiotropy (The impact of genetic variation on results is solely influenced by exposure of interest) and that all SNPs are effective tools. The fixed-effect inverse variance weighting (IVW) method was mainly used as the main analysis method (Kamiza et al., 2022). The intercept of the MR-Egger test was used to examine potential pleiotropic effects. Scatterplots are used to display the findings of several MR procedures. Odds ratios (ORs) and 95% confidence intervals (CIs) were used to represent the causal effects of overall and Melanoma. We utilized scatterplots to show the genetic relationship between glycemic characteristics and melanoma risk, and funnel plots to visually analyze the consistency of MR estimations and potential related biases. R software was used for these analyses, where the “Two-Sample MR” and “MR-PRESSO” R packages were used.

### 2.4 Sensitivity analysis

Pleiotropy was investigated using the MR-Egger approach, which was used to determine if a single locus impacts numerous phenotypes. Second, the Leave-one-out sensitivity test was used to gradually remove the SNPs to ensure that the results were credible. The Cochran Q statistic was used to standardize heterogeneity analyses. In addition, MR PRESSO was used to detect and eliminate anomalous instrumental factors.

## 3 Results

### 3.1 MR assessment of glucose traits and melanoma risk

After a quality control process, we obtained 10 SNPs strongly associated with 2-h Glucose, 47 SNPs strongly associated with Fasting Glucose, 26 SNPs associated with Fasting Insulin, and 105 strongly associated with Type-2 Diabetes from GWAS and 68 SNPs closely related to Fasting Glucose. The F-statistics of these SNPs were all greater than 10, indicating that our instrumental variables were closely related to Glucose Traits. Furthermore, our instrumental variables were not directly associated with the risk of Melanoma (Table 1).

**TABLE 1:** Power calculation for Mendelian randomization analyses for glycemic traits in relation to Melanoma risk.

Glycemic trait	Remove the SNPs for selection criteria above	Remove the SNPs for palindromic and ambiguous structure	No. of SNPs for MR	Variance explained %	F statistic	MR egger Q statistic	MR egger *p*-value
Fasting insulin	26	0	26	0.0189	55	30.24	0.1768
2-h glucose	12	2	10	0.1728	58	6.65	0.5749
Fasting glucose	53	6	47	0.0106	117	57.99	0.0926
HbA1c	76	8	68	0.0096	108	88.38	0.0344
Type-2 diabetes	105	44	105	0.1854	89	112.72	0.2409

### 3.2 Mendelian randomization analysis of the association between glycemic traits and the risk of melanoma

**FIGURE 1:**
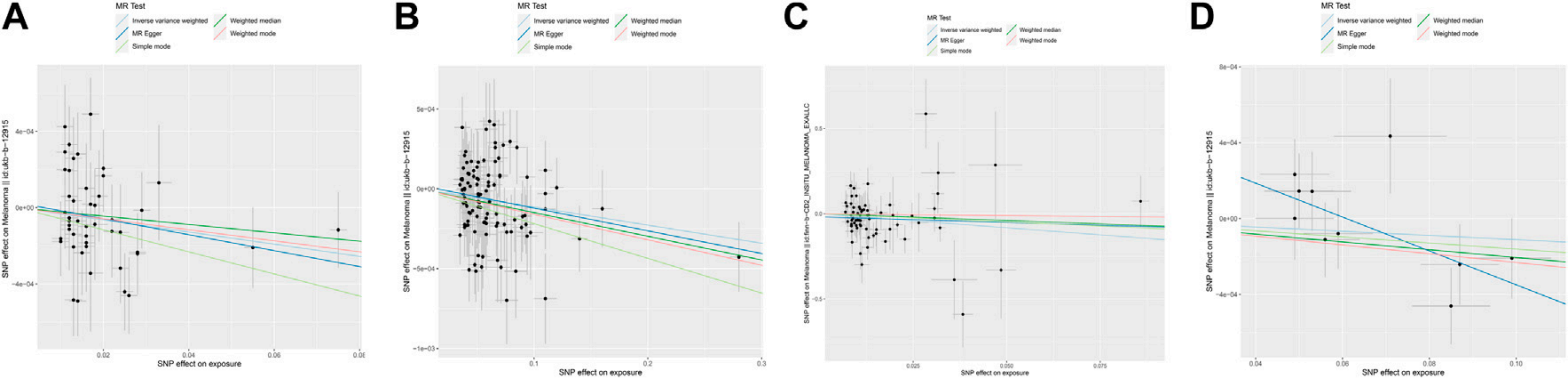
Scatter plot of genetic causality between Glycemic Traits and Melanoma using different MR methods. **(A)** Fasting Glucose **(B)** Type 2 Diabetes **(C)** HbA1c **(D)** 2-h Glucose. The dark blue line represents MR Egger, the light green line represents simple mode, the dark green line represents Weighted median, the light blue line represents IVW, and the red line represents Weighted mode.

IVW provides accurate estimates since the lack of heterogeneity and directional pleiotropy between exposure and outcome variables. Focusing primarily on the results of the IVW analysis, we assessed the causal relationship between these SPNs and melanoma risk with the Glycemic Traits (Table 2). The results showed that Fasting Glucose (OR = 0.99, 95%CI = 0.993–0.998, *p* < 0.05, IVW), Type-2 Diabetes (OR = 0.998, 95%CI = 0.998–0.999, *p* < 0.01, IVW) and HbA1c (OR= 0.19, 95%CI = 0.0415–0.8788, *p* < 0.05, IVW) was causally associated with a lower risk of Melanoma. Its orientation is consistent with several other MR analysis methods (Figures 1A–C). The 2-h Glucose and Fasting Insulin results showed no apparent causal relationship with Melanoma risk. Among them, no obvious causal difference was found in the IVW analysis of 2-h Glucose, but its *p* < 0.05 in MR Egger, and the direction was consistent in other MR analyses, suggesting that there may be a causal relationship (Figure 1D).

**TABLE 2:** Associated between the Glycemic Traits and risk of Melanoma using two-sample MR.

Glycemic traits	MR method	OR	95%CI	*p*-value
**2-Hour Glucose**				
	MR Egger	0.9911	0.9883–0.9984	<0.05
	Weighted Median	0.9980	0.9995–1.0005	0.11
	Inverse Variance Weighted	0.9989	0.9996–1.0001	0.31
	Simple Mode	0.9984	0.9994–1.0002	0.47
	Weighted Mode	0.9977	0.9984–1.0001	0.17
**Fasting Glucose**				
	MR Egger	0.9958	0.9901–1.0015	0.16
	Weighted Median	0.9978	0.9937–1.0018	0.30
	Inverse Variance Weighted	0.9968	0.9938–0.9998	<0.05
	Simple Mode	0.9942	0.9860–1.0025	0.13
	Weighted Mode	0.9971	0.9929–1.0012	0.16
**Fasting Insulin**				
	MR Egger	0.9917	0.9716–1.0121	0.43
	Weighted Median	1.0011	0.9941–1.0082	0.76
	Inverse Variance Weighted	0.9997	0.9943–1.0051	0.91
	Simple Mode	1.0032	0.9906–1.0159	0.62
	Weighted Mode	1.0025	0.9925–1.0127	0.67
**Type-2 Diabetes**				
	MR Egger	0.9986	0.9972–0.9999	<0.05
	Weighted Median	0.9985	0.9975–0.9995	<0.01
	Inverse Variance Weighted	0.9989	0.9983–0.9995	<0.01
	Simple Mode	0.9978	0.9959–0.9998	<0.05
	Weighted Mode	0.9984	0.9973–0.9996	<0.05
**HbA1c**				
	MR Egger	0.5199	0.029–9.3293	0.66
	Weighted Median	0.4510	0.0496–4.1015	0.46
	Inverse Variance Weighted	0.1910	0.0415–0.8788	<0.05
	Simple Mode	0.3793	0.004–35.9837	0.65
	Weighted Mode	0.8021	0.0863–7.4481	0.85

### 3.3 Sensitivity analysis

**FIGURE 2:**
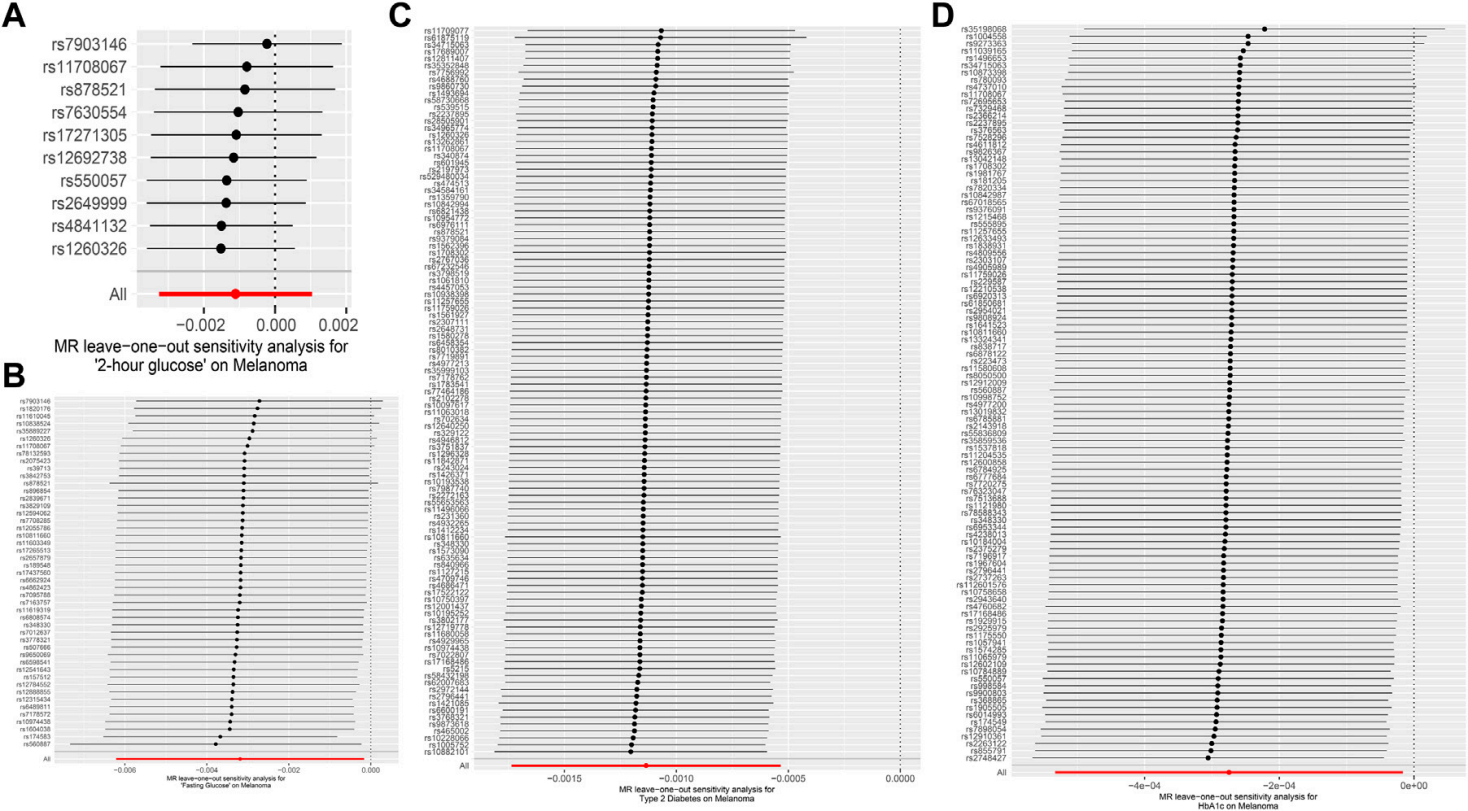
Forest map of Melanoma based on Glycemic Traits. **(A)** 2-h glucose **(B)** Fasting Glucose **(C)** Type 2 Diabetes **(D)** HbA1c. Black dots represent estimates of causal effects of Glycemic Traits on Melanoma (beta coefficients). The black line represents the estimated 95% confidence interval.

**FIGURE 3:**
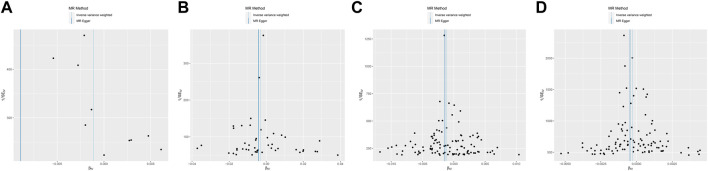
Funnel plot of Melanoma based on Glycemic Traits genetic variants. **(A)** 2-h glucose **(B)** Fasting Glucose **(C)** Type 2 Diabetes **(D)** HbA1c. Overall causal estimates (beta coefficients) of Glycemic Traits and Melanoma estimated by the IVW (light blue line) and MR-Egger (dark blue line) methods are shown.

In MR Egger, the *p*-values of MR Egger intercepts in each instrumental variable of Glycemic Traits were greater than 0.05, suggesting that the intercept does not exist, indicating fasting There was no horizontal pleiotropy for Fasting Glucose, 2-h Glucose, Fasting Insulin, HbA1c, and Type-2 Diabetes (Table 3). However, we found evidence of heterogeneity between HbA1c and melanoma risk with a *p*-value of 0.03442561 for the Q statistic. As we used the random-effects IVW as main result in MR of HbA1c, heterogeneity is acceptable (Burgess et al., 2019). No heterogeneity was found in the other analyses. Then we performed the Leave-one-out (Figures 2A–D) method and MR-PRESSO (Figures 3A–D) to identify and delete abnormal instrumental variables. The results showed that no abnormal instrumental variables were found, and the above results suggested that the MR analysis results were relatively stable.

**TABLE 3:** Estimates of Egger intercept to evaluate evidence for directional pleiotropy in MR association.

Glycemic traits	Egger intercept	SE of egger intercept	*p*-value
2-h Glucose	0.00054	0.00025	0.06
Fasting Glucose	0.00003	0.00006	0.69
Fasting Insulin	0.00013	0.00017	0.43
Type 2 Diabetes	0.00002	0.00005	0.64
HbA1c	−0.01877	0.02342	0.43

## 4 Discussion

Melanoma is a malignant tumor caused by melanocytes that is also a very deadly disease due to its high metastatic potential, accounting for 75% of skin cancer deaths (Davis et al., 2019). People who have a family history of skin cancer, have a high amount of common or underdeveloped nevi, or are excessively exposed to UV light are at high risk for melanoma (Dummer et al., 2009; Guo et al., 2016). It is still unknown whether endocrine factors can also affect the occurrence of melanoma (Guo et al., 2016). This study used the MR method for the first time to explore the causal relationship between the characteristics of Fasting Glucose (including 2-h Glucose, Fasting Glucose, Fasting Insulin, Type-2 Diabetes, and HbA1c) and the risk of Melanoma from the perspective of genetics. Overall, we found that Fasting Glucose, Type-2 Diabetes, and higher HbA1c levels were negatively associated with the risk of Melanoma. The results of MR Egger suggest that 2-h Glucose may be negatively related to the risk of Melanoma. But there is no evidence that gene-predicted Fasting Insulin levels increase the risk of Melanoma. Due to the use of suitable genetic instrument tools (F-statistics>10 and r2<0.001) in this study, no significant SNP was detected in the retention method or MR-PRESSO, and the results were highly consistent among the 5 MR algorithms. Therefore, we believe that the results of this study are to some extent reliable. The results of this study suggest that people with higher Glycemic Traits levels may be at low risk for melanoma. Patients and high-risk populations may reduce the risk of melanoma by adjusted dietary structure regulating Glycemic Traits.

In previous studies, we have noticed that Glycemic Traits exhibit different causal relationships among different tumors. There is a positive correlation between plasma glucose index and disease risk in lung cancer, but there is no significant correlation with colon cancer (Murphy et al., 2022b; Du et al., 2022). A meta-analysis shows that glycemic index will increase the overall risk of cancer, increase the risk of breast cancer (Long et al., 2022), and is positively correlated with the risk of bladder cancer and gastric cancer (Zhu et al., 2020; Kim et al., 2022). Relevant studies have shown that higher HbA1c levels are associated with an increased risk of colorectal cancer, pancreatic cancer, respiratory cancer, and female reproductive tract cancer, and are not associated with an increased risk of breast cancer, gastrointestinal or urinary system malignancies (Hong et al., 2009; Lu et al., 2015; Hope et al., 2016; Murphy et al., 2022b), but are linearly associated with overall cancer-related deaths (Yoo et al., 2022). A study shows that Type-2 Diabetes will increase the risk of colorectal cancer (Murphy et al., 2022b). The cohort study and case-control study in Melanoma suggest that Type-2 Diabetes may be negatively related to the risk of Melanoma (Harding et al., 2015; Yuan et al., 2020), but the opposite results have appeared in other studies (Qi et al., 2014; Malavolti et al., 2017). The above studies suggest that the specific relationship between Glycemic Traits and Melanoma is still contradictory.

The results of this study suggest that 2-h Glucose (OR = 0.99, 95% CI = 0.984–0.998, *p* < 0.05, MR Egger), Fasting Glucose (OR = 0.99, 95% CI = 0.993–0.998, *p* < 0.05, IVW), Type-2 Diabetes (OR = 0.998, 95% CI = 0.998–0.999, *p* < 0.01, IVW) and higher HbA1c level (OR = 0.19, 95% CI = 0.0415–0.8788, *p* < 0.05, IVW) are all possible negatively related to the risk of Melanoma. This is different from the manifestation of Glycemic Traits in causal relationships with other tumors. This may be due to the reduction of melanocytes in Diabetes patients, whose melanin content is related to plasma glucose control in diabetes and obesity (Mackiewicz-Wysocka et al., 2014). Research shows that people with light skin are about 30 times more likely to suffer from Melanoma than people with dark skin (Doepner et al., 2022). This is because the pigmentation of human skin is determined by the transfer of mature melanin synthesized by epidermal Melanoma cells to the surrounding keratinocytes. Human Chromatophores synthesize two types of melanin, namely, eumelanin (EM) and phaeomelanin (PM). The content of eumelanin is directly related to skin pigmentation and has a photoprotective effect, which can protect the skin from ultraviolet rays, thereby reducing the incidence rate of Melanoma (Upadhyay et al., 2022). Moreover, a high level of plasma glucose is often associated with high BMI and obesity. Studies have shown that obesity is associated with elevated circulating estradiol levels due to the aromatase activity of adipose tissue converting androgens into estrogen compounds (Schneider et al., 1979). In primary Melanoma, there may be high expression of estrogen receptors *β* with anti-melanoma-proliferative, and sending non-classical estrogen signals through G protein-coupled receptors (de Giorgi et al., 2013; Marzagalli et al., 2015). This may explain the different causal relationships between Glycemic Traits in Melanoma and other tumors.

Insulin is a protein hormone secreted by cells stimulated by endogenous or exogenous substances such as glucose, lactose, ribose, arginine, glucagon, *etc.* Related research suggests that it may be related to the development of tumors. Lilalutide, an analog of glucagon-like peptide 1, is a molecule that regulates glucose by increasing insulin production and inhibiting glucagon secretion. It can significantly reduce the formation of NET in tumor mice by improving the plasma glucose of patients, inhibiting tumor progression, and enhancing the anti-tumor effect of PD-1 inhibitors (Chen et al., 2022). Insulin is also an important cell growth factor that can promote cell growth, proliferation, and migration (Leitner et al., 1997). Higher fasting insulin is positively correlated with the risk of colorectal cancer (Murphy et al., 2022b). Extracellular vesicles secreted by breast cancer cells inhibit insulin secretion through miR-122, thereby damaging systemic glucose homeostasis and promoting tumor growth (Cao et al., 2022). However, in this study, the results suggest that there is no correlation between insulin level and the risk of Melanoma (OR = 0.99, 95% CI = 0.994–1.005, *p* = 0.91, IVW). Therefore, the causal relationship between Insulin and the risk of Melanoma requires more research.

There are still limitations to this study. Firstly, we restricted the relevance of the findings to other groups by concentrating on research subjects with European populations. Secondly, this study did not consider the impact of gender on MR analysis and did not conduct further subgroup analysis. Thirdly, to verify the findings, this study does not analyze additional data sources. Finally, This study used Mendelian Randomization analysis, which has the potential for weak instrument bias and pleiotropy. Future research is needed to address these limitations and to confirm the findings of this study.

Although previous observational studies can identify the relationship between Glucose, Type-2 Diabetes, Insulin and the risk of Melanoma, however, because of research confounding variables, a causal association cannot be established. In conclusion, this study used MR technology for the first time to analyze the causal relationship between Glycemic Traits and Melanoma and found that there is a negative correlation, and the underlying mechanism may provide valuable insights for carcinogenesis.

## Data Availability

The raw data supporting the conclusion of this article will be made available by the authors, without undue reservation.
