# Supplementary material for: Association between glycemic traits and melanoma: a mendelian randomization analysis
Source: Front Genet. 2023 Dec 20;14:1260367. doi: 10.3389/fgene.2023.1260367 (PMC10765500; doi:10.3389/fgene.2023.1260367)
Supplement: Supplementary file 1 [file Table1.docx]

**Supplementary Table**

| **Supplementary Table 1. Association parameters for each SNP in the glycemic traits genetic instruments** | | | | | | |
| --- | --- | --- | --- | --- | --- | --- |
| **SNP** | **effect_allele** | **other_allele** | **beta** | | ***P*-value** |  |
| **Fasting Glucose** | | | | | | |
| rs10811660 | A | G | -0.022 | 7.94E-25 | | |
| rs10838524 | A | G | 0.024 | 1.56E-40 | | |
| rs10974438 | A | C | -0.02 | 9.85E-31 | | |
| rs11603349 | T | C | 0.024 | 3.12E-25 | | |
| rs11610045 | A | G | 0.014 | 3.26E-13 | | |
| rs11619319 | A | G | -0.017 | 3.41E-20 | | |
| rs11708067 | A | G | 0.028 | 1.63E-43 | | |
| rs12055786 | T | C | 0.012 | 1.17E-11 | | |
| rs12315434 | A | C | 0.011 | 4.41E-08 | | |
| rs12541643 | T | C | 0.012 | 4.51E-09 | | |
| rs12594062 | T | C | 0.01 | 4.64E-09 | | |
| rs1260326 | T | C | -0.028 | 4.48E-65 | | |
| rs12784552 | A | G | 0.033 | 2.86E-31 | | |
| rs12888855 | A | C | -0.014 | 6.02E-12 | | |
| rs157512 | T | C | 0.013 | 5.43E-10 | | |
| rs1604038 | T | C | -0.02 | 4.47E-28 | | |
| rs17265513 | T | C | -0.016 | 5.10E-14 | | |
| rs17437560 | T | C | -0.018 | 3.33E-08 | | |
| rs174583 | T | C | -0.017 | 3.37E-22 | | |
| rs1820176 | T | C | 0.025 | 1.91E-34 | | |
| rs189548 | A | G | -0.012 | 2.81E-09 | | |
| rs2075423 | T | G | -0.016 | 3.18E-21 | | |
| rs2657879 | A | G | -0.012 | 7.33E-09 | | |
| rs2839671 | A | G | -0.016 | 8.38E-14 | | |
| rs348330 | A | G | -0.012 | 3.04E-10 | | |
| rs35889227 | T | G | -0.013 | 3.37E-10 | | |
| rs3778321 | A | G | -0.019 | 3.16E-17 | | |
| rs3829109 | A | G | -0.016 | 1.09E-15 | | |
| rs3842753 | T | G | 0.013 | 2.84E-09 | | |
| rs39713 | T | C | -0.017 | 1.77E-08 | | |
| rs4862423 | T | C | 0.012 | 4.45E-10 | | |
| rs507666 | A | G | 0.016 | 6.99E-17 | | |
| rs560887 | T | C | -0.075 | 1.00E-200 | | |
| rs6489811 | A | G | -0.011 | 3.27E-09 | | |
| rs6598541 | A | G | 0.011 | 4.12E-12 | | |
| rs6662924 | A | C | 0.014 | 3.34E-10 | | |
| rs6808574 | T | C | -0.013 | 7.21E-14 | | |
| rs7012637 | A | G | -0.018 | 9.75E-25 | | |
| rs7095788 | T | C | -0.011 | 1.98E-09 | | |
| rs7163757 | T | C | -0.022 | 2.64E-36 | | |
| rs7178572 | A | G | -0.012 | 7.09E-10 | | |
| rs7708285 | A | G | -0.013 | 1.25E-09 | | |
| rs78132593 | A | C | -0.015 | 2.60E-10 | | |
| rs7903146 | T | C | 0.026 | 2.00E-35 | | |
| rs878521 | A | G | 0.055 | 2.65E-174 | | |
| rs896854 | T | C | 0.01 | 5.61E-09 | | |
| rs9650069 | T | C | -0.029 | 8.31E-58 | | |
| **2-hour glucose** | | | | | | |
| rs11708067 | A | G | 0.087 | 1.98E-22 | | |
| rs1260326 | T | C | 0.049 | 5.93E-12 | | |
| rs12692738 | T | C | 0.049 | 2.72E-08 | | |
| rs17271305 | A | G | -0.059 | 2.88E-14 | | |
| rs2649999 | T | C | 0.05 | 2.01E-10 | | |
| rs4841132 | A | G | -0.071 | 3.13E-09 | | |
| rs550057 | T | C | 0.053 | 3.62E-11 | | |
| rs7630554 | A | G | -0.056 | 1.56E-11 | | |
| rs7903146 | T | C | 0.085 | 2.79E-26 | | |
| rs878521 | A | G | 0.099 | 1.25E-28 | | |
| **Fasting Insulin** | | | | | | |
| rs11708067 | A | G | -0.014 | 1.3E-09 | | |
| rs1206760 | A | G | -0.011 | 8.82E-10 | | |
| rs12454712 | T | C | 0.014 | 1.78E-09 | | |
| rs1260326 | T | C | -0.023 | 8.42E-38 | | |
| rs13258890 | T | C | 0.013 | 2.77E-08 | | |
| rs13389219 | T | C | -0.02 | 5.84E-28 | | |
| rs1351394 | T | C | -0.011 | 2.71E-09 | | |
| rs1474696 | A | G | -0.015 | 3.02E-16 | | |
| rs17331151 | T | C | -0.016 | 1.52E-08 | | |
| rs2108349 | A | G | -0.012 | 1.13E-08 | | |
| rs2943646 | A | G | -0.025 | 8.47E-39 | | |
| rs35000407 | T | G | 0.026 | 1.5E-21 | | |
| rs3775380 | A | G | -0.012 | 1.48E-11 | | |
| rs459193 | A | G | -0.018 | 1.12E-18 | | |
| rs4865796 | A | G | 0.017 | 7.33E-17 | | |
| rs6487237 | A | C | 0.015 | 4.68E-09 | | |
| rs6855363 | T | C | 0.013 | 4.04E-08 | | |
| rs7012814 | A | G | -0.022 | 8.34E-30 | | |
| rs7133378 | A | G | -0.013 | 6E-11 | | |
| rs73013411 | A | C | -0.018 | 2.08E-08 | | |
| rs731839 | A | G | -0.012 | 3.87E-11 | | |
| rs7903146 | T | C | -0.012 | 1.24E-09 | | |
| rs860598 | A | G | 0.018 | 6.88E-12 | | |
| rs972283 | A | G | -0.011 | 1.09E-08 | | |
| rs9819511 | T | C | 0.014 | 2.26E-08 | | |
| rs9884482 | T | C | -0.013 | 2.88E-11 | | |
| **Type 2 Diabetes** | | | | | | |
| rs1005752 | A | C | 0.079 | 5.7E-29 | | |
| rs10097617 | T | C | 0.051 | 1.1E-15 | | |
| rs10193538 | T | G | 0.037 | 1.7E-08 | | |
| rs10195252 | T | C | 0.06 | 1.6E-20 | | |
| rs10228066 | T | C | 0.066 | 1.9E-25 | | |
| rs1061810 | A | C | 0.05 | 8.5E-13 | | |
| rs10750397 | A | G | 0.045 | 2E-10 | | |
| rs10811660 | A | G | -0.16 | 6.6E-79 | | |
| rs10842994 | T | C | -0.074 | 2.5E-20 | | |
| rs10882101 | T | C | 0.11 | 1.6E-62 | | |
| rs10938398 | A | G | 0.044 | 4.9E-12 | | |
| rs10954772 | T | C | 0.041 | 2.3E-09 | | |
| rs10974438 | A | C | -0.051 | 1.6E-14 | | |
| rs11063018 | T | C | -0.053 | 1.6E-10 | | |
| rs11257655 | T | C | 0.09 | 3.7E-32 | | |
| rs1127215 | T | C | -0.047 | 2.3E-13 | | |
| rs11496066 | T | C | 0.047 | 1.2E-08 | | |
| rs11680058 | A | G | 0.058 | 1.3E-08 | | |
| rs11708067 | A | G | 0.089 | 1.3E-31 | | |
| rs11709077 | A | G | -0.11 | 1.6E-27 | | |
| rs11759026 | A | G | -0.067 | 1.3E-18 | | |
| rs11842871 | T | G | -0.042 | 1.5E-08 | | |
| rs12001437 | T | C | -0.041 | 3.7E-10 | | |
| rs1260326 | T | C | -0.067 | 1.3E-24 | | |
| rs12640250 | A | C | -0.039 | 4.5E-08 | | |
| rs12719778 | T | C | 0.039 | 2.1E-09 | | |
| rs12811407 | A | G | 0.049 | 2.4E-12 | | |
| rs1296328 | A | C | 0.035 | 4.3E-08 | | |
| rs13262861 | A | C | -0.094 | 1.8E-27 | | |
| rs1359790 | A | G | -0.083 | 5.7E-31 | | |
| rs1412234 | T | C | -0.043 | 2.5E-10 | | |
| rs1421085 | T | C | -0.12 | 2.4E-78 | | |
| rs1426371 | A | G | -0.05 | 1.1E-11 | | |
| rs1493694 | T | C | 0.084 | 2.1E-16 | | |
| rs1561927 | T | C | -0.043 | 1.9E-09 | | |
| rs1562396 | A | G | -0.058 | 7.6E-17 | | |
| rs1573090 | T | G | 0.05 | 8.4E-15 | | |
| rs1580278 | A | C | -0.041 | 2.9E-10 | | |
| rs1708302 | T | C | -0.092 | 4.2E-48 | | |
| rs17168486 | T | C | 0.069 | 6.9E-17 | | |
| rs17522122 | T | G | 0.038 | 4E-09 | | |
| rs17689007 | A | G | -0.048 | 1.7E-13 | | |
| rs1783541 | T | C | 0.061 | 1.4E-14 | | |
| rs2102278 | A | G | -0.038 | 4.5E-08 | | |
| rs2197973 | T | C | 0.035 | 4.4E-08 | | |
| rs2237895 | A | C | -0.093 | 3.6E-44 | | |
| rs2272163 | A | C | -0.037 | 1.2E-08 | | |
| rs2307111 | T | C | 0.053 | 3.3E-16 | | |
| rs231360 | T | C | 0.06 | 2.9E-19 | | |
| rs243024 | A | G | 0.058 | 4.4E-20 | | |
| rs2648731 | A | G | 0.046 | 2.1E-09 | | |
| rs2767036 | A | C | -0.039 | 2.5E-08 | | |
| rs2796441 | A | G | -0.066 | 8.5E-24 | | |
| rs28505901 | A | G | -0.076 | 2.6E-21 | | |
| rs2972144 | A | G | -0.094 | 7.9E-46 | | |
| rs329122 | A | G | 0.037 | 9.2E-09 | | |
| rs340874 | T | C | -0.068 | 5.6E-26 | | |
| rs34584161 | A | G | 0.048 | 2.9E-10 | | |
| rs34715063 | T | C | -0.076 | 3.3E-14 | | |
| rs348330 | A | G | -0.051 | 3.9E-14 | | |
| rs34965774 | A | G | 0.054 | 3.5E-09 | | |
| rs35352848 | T | C | 0.071 | 9.5E-20 | | |
| rs35999103 | T | C | 0.052 | 8.3E-09 | | |
| rs3751837 | T | C | 0.044 | 1.7E-08 | | |
| rs3768321 | T | G | 0.085 | 1.3E-26 | | |
| rs3798519 | A | C | -0.058 | 1.1E-12 | | |
| rs3802177 | A | G | -0.11 | 6.3E-55 | | |
| rs4457053 | A | G | -0.059 | 1.4E-17 | | |
| rs465002 | T | C | 0.073 | 3.8E-23 | | |
| rs4686471 | T | C | -0.06 | 3.1E-20 | | |
| rs4688760 | T | C | 0.043 | 4.5E-10 | | |
| rs4709746 | T | C | -0.056 | 5E-09 | | |
| rs474513 | A | G | 0.039 | 1E-09 | | |
| rs4929965 | A | G | 0.07 | 4.8E-25 | | |
| rs4932265 | T | C | 0.065 | 7.2E-20 | | |
| rs4946812 | A | G | -0.039 | 1E-08 | | |
| rs4977213 | T | C | -0.051 | 4.4E-14 | | |
| rs5215 | T | C | -0.07 | 2E-26 | | |
| rs529480034 | T | C | 0.058 | 2.3E-09 | | |
| rs539515 | A | C | -0.051 | 1.2E-10 | | |
| rs55653563 | A | C | 0.043 | 3.2E-09 | | |
| rs58432198 | T | C | -0.065 | 1.8E-10 | | |
| rs58730668 | T | C | 0.068 | 1E-13 | | |
| rs601945 | A | G | -0.08 | 2.7E-21 | | |
| rs61875119 | A | G | 0.28 | 1E-200 | | |
| rs62007683 | T | G | -0.037 | 3.8E-08 | | |
| rs635634 | T | C | 0.05 | 8E-10 | | |
| rs6458354 | T | C | -0.051 | 3.7E-13 | | |
| rs6600191 | T | C | 0.061 | 7E-13 | | |
| rs67232546 | T | C | 0.056 | 1.4E-12 | | |
| rs6821438 | A | G | 0.042 | 5.4E-11 | | |
| rs6976111 | A | C | 0.042 | 1.5E-08 | | |
| rs7022807 | A | G | -0.04 | 3.6E-10 | | |
| rs702634 | A | G | 0.051 | 2.1E-13 | | |
| rs7178762 | T | C | -0.039 | 7E-10 | | |
| rs7719891 | A | G | -0.04 | 2.9E-08 | | |
| rs77464186 | A | C | 0.11 | 2.3E-33 | | |
| rs7756992 | A | G | -0.14 | 3E-87 | | |
| rs7987740 | T | C | 0.036 | 4.1E-08 | | |
| rs8010382 | A | G | -0.038 | 8.1E-09 | | |
| rs840966 | A | G | 0.042 | 4.6E-11 | | |
| rs878521 | A | G | 0.057 | 1.6E-14 | | |
| rs9379084 | A | G | -0.097 | 2.3E-20 | | |
| rs9860730 | A | G | 0.055 | 7.4E-15 | | |
| rs9873618 | A | G | -0.066 | 8.5E-21 | | |
| **HbA1c** | | | | | | |
| rs10405535 | A | G | 0.0122 | 6.474E-14 | | |
| rs10774624 | A | G | 0.0093 | 4.17E-14 | | |
| rs10811661 | T | C | 0.0128 | 1.735E-14 | | |
| rs11039154 | T | C | -0.0087 | 3.105E-09 | | |
| rs11248914 | T | C | 0.0114 | 1.42E-14 | | |
| rs11257655 | T | C | 0.011 | 1.911E-13 | | |
| rs112578089 | A | G | -0.036 | 2.18E-11 | | |
| rs11558471 | A | G | 0.0151 | 3.381E-25 | | |
| rs11643024 | A | G | 0.0084 | 7.975E-10 | | |
| rs11719201 | T | C | -0.0129 | 2.431E-18 | | |
| rs117233107 | A | G | -0.047 | 8.453E-11 | | |
| rs1175549 | A | C | 0.0098 | 7.127E-13 | | |
| rs12351997 | T | C | -0.0131 | 4.499E-14 | | |
| rs12491937 | A | G | 0.009 | 1.417E-13 | | |
| rs12612492 | T | C | 0.0188 | 1.883E-26 | | |
| rs1278769 | A | G | -0.0091 | 5.515E-12 | | |
| rs13134327 | A | G | 0.0144 | 2.808E-26 | | |
| rs13234131 | A | G | -0.0113 | 2.061E-09 | | |
| rs13419326 | A | C | -0.0317 | 5.6E-13 | | |
| rs1367173 | T | C | -0.0152 | 1.66E-14 | | |
| rs1535464 | A | G | -0.0086 | 1.111E-08 | | |
| rs1604038 | T | C | -0.0108 | 2.759E-16 | | |
| rs17037289 | A | G | -0.0089 | 2.402E-09 | | |
| rs174559 | A | G | -0.0106 | 3.311E-13 | | |
| rs17476364 | T | C | 0.0858 | 1E-200 | | |
| rs17533945 | T | C | -0.0128 | 1.617E-23 | | |
| rs1800562 | A | G | -0.0383 | 2.327E-50 | | |
| rs1948759 | A | G | -0.0097 | 2.442E-08 | | |
| rs2143923 | A | G | 0.0069 | 3.967E-08 | | |
| rs2273475 | A | G | -0.0128 | 1.991E-09 | | |
| rs2375278 | A | G | 0.0112 | 1.049E-11 | | |
| rs267738 | T | G | 0.0109 | 1.141E-11 | | |
| rs2748427 | A | G | -0.0307 | 9.819E-49 | | |
| rs28671200 | T | G | 0.0086 | 1.56E-08 | | |
| rs2954021 | A | G | -0.007 | 1.918E-10 | | |
| rs2971670 | T | C | 0.0316 | 5.1E-88 | | |
| rs34664882 | A | G | -0.0485 | 4.815E-37 | | |
| rs360140 | A | C | -0.0084 | 9.622E-13 | | |
| rs3778321 | A | G | -0.0106 | 4.18E-11 | | |
| rs3829109 | A | G | -0.0086 | 2.683E-08 | | |
| rs3842753 | T | G | 0.0075 | 3.933E-08 | | |
| rs452306 | T | C | -0.0098 | 5.506E-13 | | |
| rs4731113 | T | C | 0.0196 | 4.904E-08 | | |
| rs4737009 | A | G | 0.0228 | 8.292E-56 | | |
| rs4760682 | A | C | 0.0164 | 3.197E-20 | | |
| rs4980325 | T | G | 0.0108 | 4.703E-14 | | |
| rs560887 | T | C | -0.0307 | 5.55E-122 | | |
| rs61750929 | T | C | -0.0284 | 9.486E-24 | | |
| rs649129 | T | C | 0.0108 | 3.28E-15 | | |
| rs6877043 | T | C | 0.0085 | 1.987E-10 | | |
| rs6929796 | A | G | -0.0092 | 3.256E-08 | | |
| rs6931514 | A | G | -0.0102 | 1.181E-13 | | |
| rs6980507 | A | G | 0.0109 | 8.153E-20 | | |
| rs7042939 | A | G | 0.0102 | 1.497E-15 | | |
| rs7190771 | A | G | 0.0085 | 6.021E-11 | | |
| rs7198799 | T | C | 0.0083 | 4.759E-09 | | |
| rs737092 | T | C | -0.0073 | 7.569E-09 | | |
| rs7534795 | T | C | 0.01 | 2.129E-09 | | |
| rs7547793 | A | C | -0.0118 | 6.611E-09 | | |
| rs76533333 | A | G | -0.0265 | 2.81E-29 | | |
| rs7903146 | T | C | 0.0133 | 1.044E-22 | | |
| rs837763 | T | C | 0.0176 | 5.197E-38 | | |
| rs855791 | A | G | 0.0188 | 1.337E-56 | | |
| rs857725 | T | G | -0.0208 | 5.426E-55 | | |
| rs9376090 | T | C | 0.0247 | 1.897E-62 | | |
| rs9818758 | A | G | 0.0131 | 1.494E-13 | | |
| rs9909940 | T | C | 0.0322 | 1.43E-116 | | |
| rs9914988 | A | G | 0.0125 | 4.656E-17 | | |

Chen, Ji, Cassandra N. Spracklen, Gaëlle Marenne, Arushi Varshney, Laura J. Corbin, Jian'an Luan, Sara M. Willems*, et al.* "The Trans-Ancestral Genomic Architecture of Glycemic Traits." [In eng]. *Nature Genetics* 53, no. 6 (2021): 840-60. <https://doi.org/10.1038/s41588-021-00852-9>. <https://pubmed.ncbi.nlm.nih.gov/34059833>.

Mahajan, Anubha, Daniel Taliun, Matthias Thurner, Neil R. Robertson, Jason M. Torres, N. William Rayner, Anthony J. Payne*, et al.* "Fine-Mapping Type 2 Diabetes Loci to Single-Variant Resolution Using High-Density Imputation and Islet-Specific Epigenome Maps." [In eng]. *Nature Genetics* 50, no. 11 (2018): 1505-13. <https://doi.org/10.1038/s41588-018-0241-6>. <https://pubmed.ncbi.nlm.nih.gov/30297969>.
